# Integration of mRNA and miRNA Analysis Reveals the Regulation of Salt Stress Response in Rapeseed (*Brassica napus* L.)

**DOI:** 10.3390/plants14152418

**Published:** 2025-08-04

**Authors:** Yaqian Liu, Danni Li, Yutong Qiao, Niannian Fan, Ruolin Gong, Hua Zhong, Yunfei Zhang, Linfen Lei, Jihong Hu, Jungang Dong

**Affiliations:** 1 State Key Laboratory of Crop Stress Resistance and High-Efficiency Production, College of Agronomy, Northwest A&F University, Yangling 712100, China; 2 Population Sciences in the Pacific Program, University of Hawai‘i at Mānoa, Honolulu, HI 96813, USA

**Keywords:** *Brassica napus* L., salt stress, RNA-Seq, miRNA, posttranscriptional regulation, qRT-PCR

## Abstract

Soil salinization is a major constraint to global crop productivity, highlighting the need to identify salt tolerance genes and their molecular mechanisms. Here, we integrated mRNA and miRNA profile analyses to investigate the molecular basis of salt tolerance of an elite *Brassica napus* cultivar S268. Time-course RNA-seq analysis revealed dynamic transcriptional reprogramming under 215 mM NaCl stress, with 212 core genes significantly enriched in organic acid degradation and glyoxylate/dicarboxylate metabolism pathways. Combined with weighted gene co-expression network analysis (WGCNA) and RT-qPCR validation, five candidate genes (*WRKY6*, *WRKY70*, *NHX1*, *AVP1*, and *NAC072*) were identified as the regulators of salt tolerance in rapeseed. Haplotype analysis based on association mapping showed that *NAC072*, *ABI5*, and *NHX1* exhibited two major haplotypes that were significantly associated with salt tolerance variation under salt stress in rapeseed. Integrated miRNA-mRNA analysis and RT-qPCR identified three regulatory miRNA-mRNA pairs (bna-miR160a/*BnaA03.BAG1*, novel-miR-126/*BnaA08.TPS9*, and novel-miR-70/*BnaA07.AHA1*) that might be involved in S268 salt tolerance. These results provide novel insights into the post-transcriptional regulation of salt tolerance in *B. napus*, offering potential targets for genetic improvement.

## 1. Introduction

Rapeseed, as a globally significant oil crop, holds extremely high production value. Driven by climate change and a rising population, rapeseed oil production has surged from 154.2 Mt in 1979 to 72.37 Gt in 2020, becoming the second-largest oil crop after soybeans and a key focus for yield optimization due to its dual use in vegetable oil and biofuel [1]. Some studies suggest that rapeseed has advantages such as strong salt–alkali tolerance, low planting costs, and rapid growth, making it a pioneer crop for the improvement and utilization of saline–alkali land [2]. This characteristic enables it to be successfully cultivated in salt-affected areas, demonstrating good environmental adaptability.

Salinity, as a major environmental stress factor, affects crop production in many regions globally. It is reported that approximately 1 billion hectares are currently salt-affected, accounting for about 7% of the Earth’s land surface. In China, salinized land constitutes around 5.01% of the national territory, posing a significant threat to food security [3]. It is expected that the affected area will expand significantly, and economic losses may continue to increase [4]. Soil salinization can affect seed germination and photosynthesis, and diminish crop yield and quality, leading to a reduction in arable land area [5]. The adverse impact of salinization on plants results from the reduction of water availability in saline soil and the toxic effect of excessive accumulation of Na^+^ and Cl^−^, as well as a lack of oxygen (O_2_) underground [6]. It is well known that salinity induces alterations in metabolic activities, causes damage to the cell wall, and leads to cytoplasmic dissolution. It diminishes photosynthetic efficiency, accelerates senescence, increases respiratory consumption, and promotes the accumulation of toxins, ultimately resulting in plant mortality [7]. However, plants are not completely passive. They respond and adapt to salt stress by rapidly inducing changes at the transcription, post-transcription, translation, and post-translational levels, thus providing mechanisms that enable the development of salt-tolerant crops [8]. Consequently, plants have to adjust their growth and developmental paradigms to restore osmotic and ionic homeostasis, detoxifying secondary damage under salt stress [9]. Transcriptome analysis has revealed the genes associated with carbohydrate metabolism, hormone, and MAPK signaling pathways involved in salt tolerance in rapeseed [10]. Exploring the molecular regulatory mechanisms of salt-tolerant rapeseed varieties plays an important role in the stable yield of rapeseed and in the effective utilization of salinized lands.

Small RNAs are essential regulators of gene expression in crops at the transcriptional and post-transcriptional levels [11]. MicroRNAs (miRNAs) are endogenous, short (18–24 nt), non-coding RNA molecules that negatively regulate gene expression [12]. Previous reports have shown that miRNAs are pivotal regulators of salt stress tolerance in diverse plant species. In *Oryza sativa* L., miR1861h is transcriptionally upregulated in response to salt stress, and its overexpression markedly enhances salt tolerance, as demonstrated in transgenic lines derived from Dongxiang wild rice [13]. In contrast, miR417 exerts a negative regulatory effect on salt stress tolerance in *Arabidopsis thaliana* by inhibiting seed germination under high salinity conditions, although its specific target genes remain to be conclusively identified [14]. In cotton, a new microRNA, miRNVL5, is associated with increased salt sensitivity, and plants overexpressing miRNVL5 in *A. thaliana* exhibit hypersensitivity to salt stress [15]. Furthermore, miR402 in *A. thaliana* modulates salt stress responses by targeting the DNA demethylase DML3, thereby influencing DNA methylation patterns and subsequent downstream signaling pathways [16]. It has been demonstrated that miRNA plays important roles in plant salinity responses and adaptation through various miRNA-mediated biological processes, including signal transduction, membrane transport, protein biosynthesis and degradation, photosynthesis, and transcription [17].

The expression profiles of miRNAs and mRNAs associated with salt tolerance in many plants were explored by combining miRNA-seq and RNA-seq to determine their roles in regulation, including cotton (*Gossypium hirsutum* L.) [18], potato (*Solanum Tuberosum* L.) [19], sugar beet (*Beta Vulgaris* L.) [20], tobacco (*Nicotiana tabacum* L.) [21], and day lily (*Hemerocallis fulva*) [22]. Plant miRNA regulatory modules play critical roles in salt stress tolerance: for instance, overexpression of the miR156–SPL–DFR pathway enhances salt tolerance in transgenic *Arabidopsis* [23], while in cotton, elevated miR160b levels or suppression of its targets *GhARF17/18* promote development and root biomass under salt stress [24]. Moreover, the miR171/miR172 module improves salt tolerance in soybean, *Arabidopsis*, and rice by downregulating targets such as SSAC1 and IDS1 [25,26,27,28]. In contrast, salt stress and ABA induce the miR394–LCR module, with overexpression of miR394a/b or loss of LCR function resulting in heightened salt sensitivity [29]. However, studies of miRNA regulatory mechanisms in salt-tolerant rapeseed cultivars remain scarce. In this study, we identified a highly salt-tolerant rapeseed cultivar, Shaanyou 268 (S268), and conducted RNA-seq and miRNA-seq analysis to reveal the conserved and novel miRNAs and the regulatory roles of miRNAs with their targets under salt stress. These findings on the miRNA-mRNA regulatory networks will provide insights into the molecular mechanisms underlying the regulation of salt tolerance in rapeseed.

## 2. Results

### 2.1. Evaluation of Salt Stress Tolerance in Different Rapeseed Cultivars

**Figure 1:**
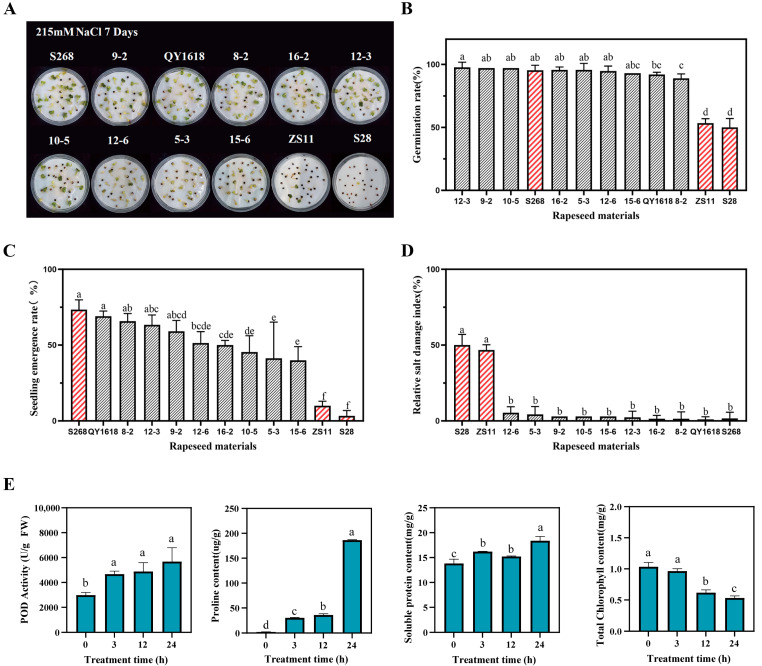
Comparison of phenotypes, seed germination, and seedling growth of the rapeseed accessions under salt stress. (**A**) Phenotypes of the rapeseed accessions after 7 days of exposure to 215 mM NaCl stress; (**B**) Germination rate, (**C**) Seedling rate, and (**D**) Relative salt damage index of the rapeseed accessions. (**E**) Physiological responses of S268 under salt stress (0 h, 3 h, 12 h, and 24 h), including peroxidase (POD) activity, proline content, soluble protein content, and total chlorophyll content. Error bars in the figure represent the standard deviation (SD) of the mean (*n* = 3). Lowercase letters indicate significant differences at *p* < 0.05.

Based on seed germination, seedling growth, and relative salt damage index, this study systematically evaluated 88 rapeseed cultivars, including Zhongshuang 11 (ZS11) and S268 (Table S1). The results showed that rapeseed cultivar S268 exhibited relatively high salt tolerance, being significantly higher than those of other cultivars (Figure 1). To further elucidate salt-tolerance phenotypic characteristics, phenotypic analyses and relative salt damage index comparisons were conducted (Figure 1B–D). The results suggested that rapeseed cultivar S268 displayed significant growth advantages under salt stress conditions, with a relatively low salt damage index under 215 mM NaCl stress (Figure 1D).

To characterize the physiological changes in S268 under NaCl stress, we measured changes in proline content, POD activity, soluble protein content, and total chlorophyll content 0, 3, 12, and 24 h after salt stress treatment (Figure 1E). The results showed that proline content increased continuously with the duration of stress. POD activity increased significantly in the early stages of stress and reached its peak, which was significantly higher than the initial level, at 24 h. Soluble protein content was significantly higher at 3 and 12 h compared to the control, and further increased at 24 h. Total chlorophyll content remained relatively stable during the early stages of stress but showed a moderate decline at 12 and 24 h. Collectively, these physiological responses demonstrated that S268 exhibits relatively strong salt tolerance by maintaining effective osmotic adjustment, antioxidant defense, and photosynthetic capacity under salt stress conditions.

### 2.2. Functional Enrichment and Expression Dynamics of Core Genes in Rapeseed Cultivar S268 Under Salt Stress

In order to identify the differentially expressed genes (DEGs) in response to salt stress in rapeseed cultivar S268, RNA-seq of three independent biological replicates was performed after 0 h (control), 3 h, 12 h, and 24 h of exposure to NaCl stress. In this study, transcriptome sequencing of 12 samples was successfully conducted, yielding a total of 271.11 million reads and accumulating 80.73 gigabases (Gb) of clean data. The average data volume per sample was 6.73 Gb, with the percentage of Q30 bases reaching 97.91% or higher. Each sample contributed 6.00 Gb of clean data, culminating in a collective total of 72 Gb of clean data with a minimum Q30 quality score of 85% (Table S2).

**Figure 2:**
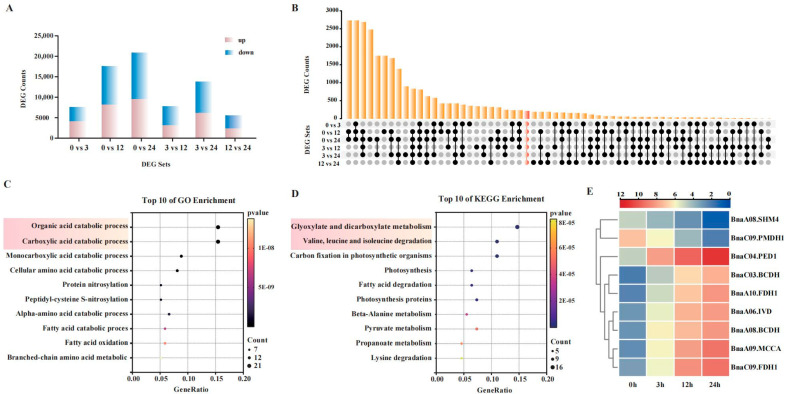
Functional enrichment and expression analysis of core genes in rapeseed S268 under salt stress. (**A**) Distribution of DEGs at different salt stress time points; (**B**) comparison of overlapping genes among salt stress time groups, the bar chart above indicates the number of DEGs in each overlapping set. The connected black circles below represent the specific groups involved in each intersection, with gray circles indicating absence and black circles indicating presence; (**C**,**D**) GO and KEGG enrichment analysis of 212 common genes across six temporal comparison groups; (**E**) clustered heatmap showing expression patterns of 9 common genes in significant GO and KEGG pathways.

Transcriptomic analysis of the salt-tolerant rapeseed cultivar S268 grown under 215 mM NaCl stress revealed dynamic changes in DEGs over prolonged stress durations (Figure 2A). The number of DEGs exhibited a significant time-dependent increase compared to the non-stressed condition (0 h): 7593 DEGs (4094 upregulated, 3499 downregulated) were detected at 3 h, rising to 17,565 DEGs (8166 upregulated, 9399 downregulated) at 12 h, and peaking at 20,917 DEGs (9565 upregulated, 11,352 downregulated) by 24 h. This trend indicates a cumulative regulatory effect of salt stress on gene expression in rapeseed (Table S3).

Comparative analysis of DEGs between adjacent time points revealed phase-specific characteristics in transcriptional dynamics. A total of 7781 DEGs were identified from 3 h to 12 h (59.7% downregulated), likely associated with the transition from early stress signaling to homeostatic adaptation. In contrast, the number of DEGs decreased to 5506 (57.9% downregulated) between 12 h and 24 h, reflecting stabilization of regulatory mechanisms in later phases. Notably, 13,823 DEGs (55.6% downregulated) were observed between 3 h and 24 h, highlighting systemic reprogramming of gene expression patterns under prolonged stress.

To compare DEGs across distinct salt-stress time points (0, 3, 12, and 24 h), this study conducted an intersection analysis of DEGs from six comparison groups (0 vs. 3, 0 vs. 12, 0 vs. 24, 3 vs. 12, 3 vs. 24, and 12 vs. 24 h) (Figure 2B). A total of 212 genes were identified as consistently differentially expressed across all time points, suggesting these core genes may play persistent and critical roles in salt-stress responses. Gene Ontology (GO) enrichment analysis revealed that the 212 salt-tolerant core genes were significantly enriched in organic acid catabolic processes (GO:0016054, *p*-value = 1.13 × 10^−18^) and carboxylic acid catabolic processes (GO:0046395, *p*-value = 1.13 × 10^−18^), with complete overlap of genes between these two pathways (Figure 2C). Furthermore, KEGG enrichment analysis demonstrated significant enrichment of these core genes in glyoxylate and dicarboxylate metabolism (ko00630, *p*-value = 4.88 × 10^−13^) and branched-chain amino acid degradation (ko00280, *p*-value = 9.65 × 10^−11^) (Figure 2D and Table S4).

Analysis of the expression patterns of nine core genes co-enriched in both GO pathways (GO:0016054 and GO:0046395) and KEGG pathways (ko00280 and ko00630) revealed a spatiotemporal regulatory strategy underlying salt tolerance in *B. napus* (Figure 2E and Tables S4 and S5). Genes associated with branched-chain amino acid degradation (e.g., *BnaC04.PED1*, upregulated 72.4-fold) were persistently induced, channeling carbon skeletons into the TCA cycle to drive ATP-dependent ion transport. Conversely, glyoxylate cycle genes (e.g., *BnaA08.SHM4*, downregulated 33.3-fold) were suppressed early, redirecting carbon flux toward gluconeogenesis. Notably, *BnaC09.FDH1* (upregulated 75.8-fold) synergized with *BnaA10.FDH1* to alleviate formation toxicity, thereby supporting mitochondrial energy production. The dynamic remodeling of carbon–nitrogen metabolism and phase-specific antioxidant regulation (e.g., *BnaC09.PMDH1*) demonstrated that temporal coordination of catabolic pathways is critical for osmotic adjustment, redox homeostasis, and resource allocation under salt stress. This study suggests that these metabolic pathways might be involved in balancing stress defense and energy homeostasis.

**Figure 3:**
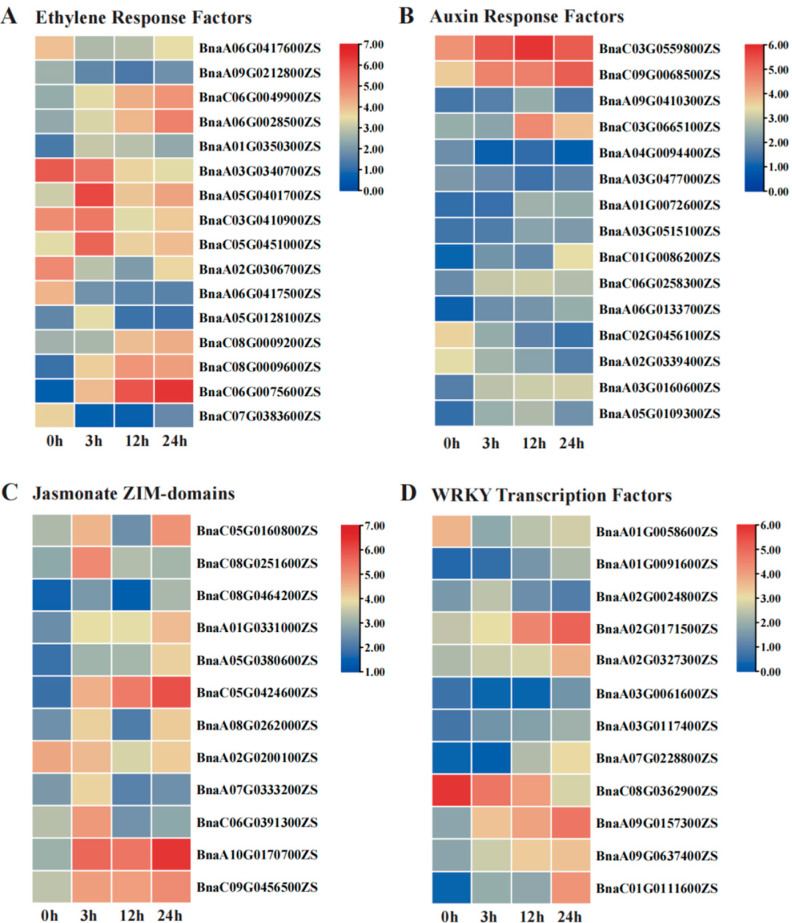
Analysis of differential gene expression patterns involved in phytohormone pathways and transcription factors in *Brassica napus* S268 at different time points under salt stress. Differentially expressed genes (DEGs) involved in (**A**) ethylene, (**B**) auxin, and (**C**) jasmonic acid (JA) signaling pathways; (**D**) changes in expression patterns of *WRKY* genes at different salt stress time points.

Transcriptome profiling of the *Brassica napus* cultivar S268 under salt stress (0 h, 3 h, 12 h, and 24 h) revealed the dynamic expression patterns of key transcriptional regulators. Some of the DEGs were involved in phytohormone signaling pathways, including auxin, ethylene, and jasmonic acid (JA) (Figure 3). Under salt stress, *BnaA01.ERF4* (*BnaA01G0350300ZS*) was rapidly induced at 3 h (4.6-fold), whereas *BnaC06.ERF3* (*BnaC06G0049900ZS*) showed sustained upregulation (5.7-fold from initial values at 24 h). Functional divergence was evident as *ERF4* homologs (*BnaA03G0340700ZS* versus *BnaA01G0350300ZS*) exhibited opposing trends, highlighting neofunctionalization post-duplication (Figure 3A). Meanwhile, for the auxin signaling pathway, at 3 h, *BnaC03.ARF2* (*BnaC03G0559800ZS*) doubled from 20.84 to 40.51 (FPKM value), while by 12 h, miR167-regulated *BnaC03.ARF6* (*BnaC03G0665100ZS*) increased 5-fold from 4.62 to 22.96 (FPKM value) (Figure 3B). Long-term adaptation involved *BnaA06.ARF19* (*BnaA06G0133700ZS*), which maintained high expression and covaried with ethylene signaling, whereas *BnaA04.ARF8* (*BnaA04G0094400ZS*) was suppressed. The *JAZ* genes demonstrated three dynamic modes: *BnaC05G0160800ZS* exhibited pulse-like activation (2.4-fold at 24 h), while *BnaC05.JAZ3* (*BnaC05G0424600ZS*) showed sustained 22.9-fold upregulation from 0 h to 24 h (Figure 3C). Among the WRKY transcription factors, eleven genes exhibited differential expression patterns with two dominant regulatory modes (Figure 3D). For instance, *BnaA02.WRKY9* (*BnaA02G0171500ZS*) showed sustained upregulation (7.2-fold) over 24 h, while *BnaC08.WRKY70* (*BnaC08G0362900ZS*) was suppressed, suggesting the WRKY transcription factors were involved in the salt stress response. Collectively, these findings indicate a hierarchical regulatory framework that might mediate growth–stress trade-offs via temporally specific activation of transcription factors and integration across multiple signaling pathways.

### 2.3. Co-Expression Network Analysis and Time Point-Specific Modules in Rapeseed S268 Under Salt Stress

**Figure 4:**
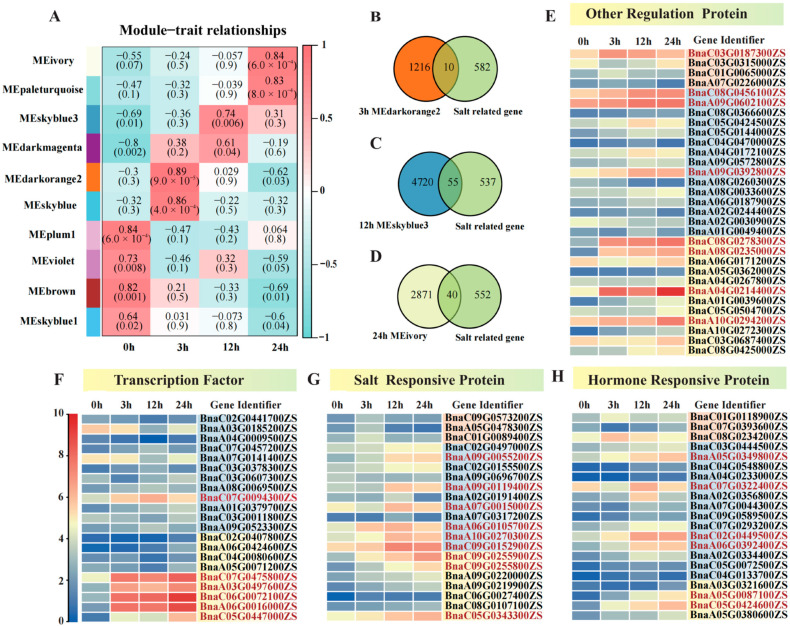
Weighted gene co-expression network analysis (WGCNA) and differentially expressed genes (DEGs) of modules involved in salt stress. (**A**) Characteristics of co-expression network modules under salt stress. (**B**–**D**) Overlapping DEGs between salt stress-related homologous genes and MEdarkorange2 module in 3 h. (**B**) MEskyblue3 module at 12 h. (**C**) MEivory module at 24 h (**D**). (**E**–**H**) Expression patterns of DEGs in regulatory pathways: (**E**) Other regulation; (**F**) Transcription factors (TFs); (**G**) Salt response; and (H) phytohormone signaling pathway. Color intensity represents expression levels, with red indicating high expression and blue indicating low expression.

To further investigate the regulatory mechanisms of S268 at different time points (3 h, 12 h, 24 h) of salt stress, we constructed co-expression networks across distinct salt stress time points into 10 modules using Weighted Gene Co-Expression Network Analysis (WGCNA). Module–trait association analysis found that three modules—MEdarkorange2, MEskyblue3, and MEivory—showed significant correlations with the 3 h, 12 h, and 24 h salt stress time points, respectively. Specifically, the 3 h-associated MEdarkorange2 module contained 1226 DEGs, representing an early stress-responsive gene set. The 12 h-associated MEskyblue3 module included 4775 DEGs, and the 24 h-associated MEivory module comprised 2911 DEGs, indicating that these DEGs were involved in salt stress response (Figure 4A and Figure S1).

GO enrichment analysis of the salt stress 3 h-specific response module MEdarkorange2 identified via WGCNA revealed that genes in this module were significantly enriched for association with early responses to salt stress (Figure S1). “Hyperosmotic salinity response” (GO:0042538) and “Hyperosmotic response” (GO:0006972) exhibited the highest enrichment levels as core terms, indicating that this module may regulate osmotic adjustment mechanisms to counteract the hyperosmotic environment during the initial phase of salt stress. Notably, “Abscisic acid metabolism” (GO:0009687), “Abscisic acid biosynthetic process” (GO:0009688), and “Apocarotenoid biosynthetic process” (GO:0043289) were also enriched (Figure S1A). Importantly, KEGG enrichment analysis demonstrated systemic activation of secondary metabolite synthesis pathways, including “Carotenoid biosynthesis” (ko00906) and “Steroid biosynthesis” (ko00100). These findings suggest that the synthesis of ABA precursors may be driven by genes in this module (Figure S1B), highlighting the potential pivotal role of the ABA signaling pathway in the early salt stress response. Based on these results, we hypothesize that *B. napus* S268 coordinates cellular osmotic protection and hormonal regulatory networks during the early phase of salt stress (3 h) by activating osmotic adjustment mechanisms, initiating ABA signaling, and promoting the synthesis of secondary metabolites such as carotenoids, thereby establishing an integrated defense strategy against initial salt stress (Table S5).

Based on the GO and KEGG enrichment analyses of the 12 h salt stress-specific module MEskyblue3, the significantly enriched “Vesicle fusion” (GO:0006906) and “Membrane fusion” (GO:0061025) pathways in GO (Figure S1C) were directly linked to the highly significant “SNARE interactions in vesicular transport “ (ko04130) in KEGG (Figure S1D). The SNARE protein-mediated membrane fusion mechanism may synergistically maintain the integrity of organelle membrane systems by regulating “Vacuolar transport” (GO:0007034) and “Peroxisomal transport” (GO:0043574), thereby alleviating osmotic imbalance caused by salt stress, suggesting their critical role in oxidative metabolism and ROS scavenging during salt stress. Thus, it is indicated that under 12 h salt stress, *B. napus* S268 may dynamically remodel organelle membrane systems (e.g., vacuoles and peroxisomes) through SNARE-mediated vesicular transport, synergistically enhancing ROS clearance to maintain osmotic balance and redox homeostasis, representing a core salt-tolerance strategy (Table S5).

Using WGCNA analysis, the gene co-expression characteristics and biological functions of the MEivory module in the S268 under 24 h salt stress were revealed. GO enrichment analysis showed that salicylic acid (SA)-related pathways exhibited the strongest enrichment signals. Specifically, “Salicylic acid metabolic process” (GO:0009696), “Salicylic acid biosynthesis” (GO:0009697), and “Salicylic acid metabolic regulation” (GO:0010337) are enriched, indicating that SA signaling plays a central regulatory role in long-term salt stress responses (Figure S1E). Concurrently, KEGG analysis revealed the co-activation of “Glutathione metabolism” (ko00480) and “Ascorbate metabolism” (ko00053) (Figure S1F). As a systemic resistance signaling molecule, SA may enhance reactive oxygen species (ROS) scavenging capacity by regulating the expression of antioxidant enzyme genes such as glutathione peroxidase (GPX) (GO:0010112). Furthermore, the association between “Jasmonic acid signaling regulation” (GO:2000022) and “Alpha-linolenic acid metabolism” (ko00592) in KEGG suggests that the biosynthesis of “Alpha-linolenic acid”, a JA precursor, may activate JA signaling cascades through the lipoxygenase pathway, suggesting SA-JA cross-talk to coordinate stress resistance (Figure S1E,F; Table S5).

In our study, *B. napus* S268 dynamically coordinates salt stress responses through time-specific regulatory strategies. During the initial 3 h salt exposure period, the plant activates osmotic adjustment mechanisms and ABA signaling pathways, potentially mediated by carotenoid biosynthesis. Along with the time increasing (12 h), cellular defense shifts to rely on vesicle transport-mediated peroxisome ROS scavenging systems. Finally, after 24 h of salt stress, the response transitions to a synergistic SA-JA regulatory network, engaging glutathione and ascorbate metabolic pathways to establish a hierarchical adaptation strategy (Figure S1E,F). This phased reprogramming might highlight the molecular mechanisms of salt tolerance *in* rapeseed S268.

**Figure 5:**
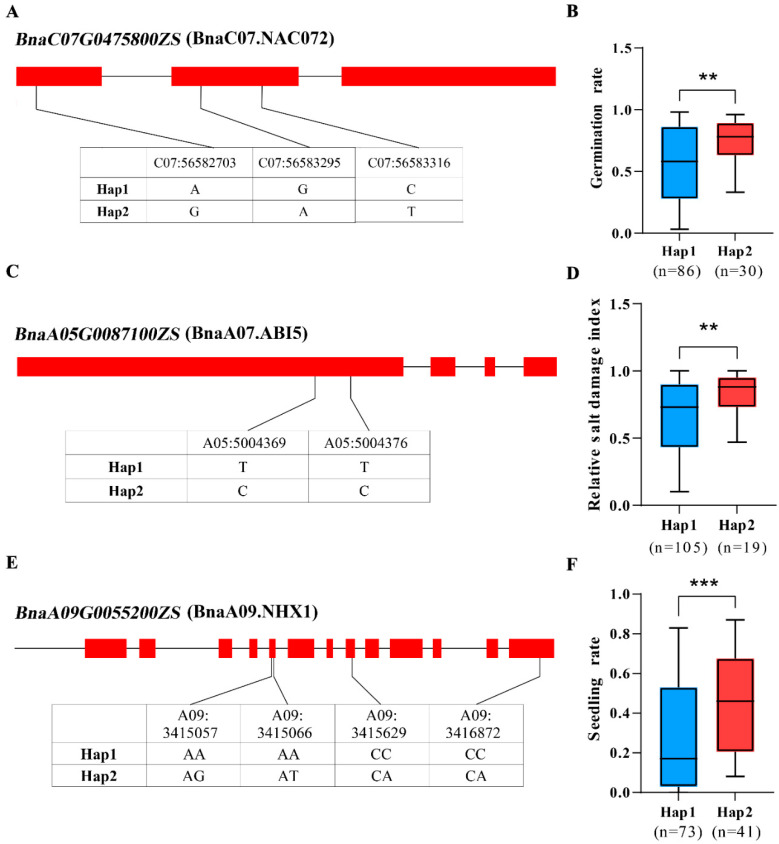
Haplotype analysis of the three candidate genes associated with salt tolerance in rapeseed. (**A**) Gene structure and the major haplotypes of *BnaC07.NAC072*. (**B**) Boxplots of germination rate under salt stress based on the haplotypes of *BnaC07.NAC072*. (**C**) Gene structure and the major haplotypes of *BnaA05.ABI5*. (**D**) Boxplots of relative salt damage index under salt stress based on the haplotypes of *BnaA05.ABI5*. (**E**) Gene structure and the major haplotypes of *BnaA09.NHX1*. (**F**) Boxplots of relative salt damage index under salt stress based on the haplotypes of *BnaA09.NHX1*. Significant differences between the haplotypes were evaluated using a two-tailed *t*-test, and the results are represented using *p*-values. ** represents *p* < 0.01, *** represents *p* < 0.001.

In these identified module-relative genes, some of the salt-stress reported response homologous genes were identified in rapeseed S268 after salt stress (Figure 4). Since few salt tolerance genes have been reported in rapeseed, these genes may be the candidate salt response genes in rapeseed. They include *BnaC08.TPS9* (*BnaC08G0278300ZS*), *BnaC07.NAC072* (*BnaC07G0475800ZS*), *BnaA07.AHA1* (*BnaA07G0015000ZS*), *BnaA09.NHX1* (*BnaA09G0055200ZS*), *BnaC09.NHX7/SOS1* (*BnaC09G0255900ZS* and *BnaC09G0255800ZS*), *BnaA05.ABI5* (*BnaA05G0087100ZS*), and *BnaC05.TIFY6B* (*BnaC05G0424600ZS*). Further association mapping and haplotype analysis in 125 rapeseed accessions revealed that the three candidate genes (*BnaC07.NAC072*, *BnaA05.ABI5,* and *BnaA09.NHX1*) were associated with the salt relative traits under salt stress, suggesting that these genes might be the causative genes for salt tolerance in rapeseed (Figure 5). The results revealed the molecular basis by which conserved modular functional units orchestrate multi-phase stress adaptation through dynamic collaboration, providing insights into salt tolerance in rapeseed.

Among the 29 genes exhibiting persistent salt stress responses (Figure 4), six genes (*TIFY6B*, *ABI5*, *TPS9*, *RCD1*, and *NHX7/SOS1*) in rapeseed showed significant enrichment in the 24 h MEivory module (Table 1), spanning critical functions such as JA signaling regulation, seed germination suppression, trehalose metabolism, and ROS homeostasis. *BnaC05.TIFY6B* (*BnaC05G0424600ZS*) was prominently associated with the JA signaling pathway (GO:2000022), bolstering abiotic stress defense. Its sustained upregulation under salt stress likely activates downstream antioxidant and ion homeostasis genes, forming a frontline defense against salt-induced damage. *BnaA05.ABI5* (*BnaA05G0087100ZS*), a transcription factor linked to “Seed germination regulation” (GO:0010029) and “Seedling development regulation” (GO:1900140), may protect early developmental stages in high-salt conditions by suppressing germination and slowing growth, a mechanism potentially contributing to salt tolerance in rapeseed cultivar S268. Concurrently, *BnaC08.TPS9* (*BnaC08G0278300ZS*) encoded trehalose-6-phosphate synthase, which synthesizes the osmoprotectant trehalose to shield membrane structures and enzymes from ionic toxicity. Meanwhile, *BnaC05.RCD1* (*BnaC05G0343300ZS*) and *BnaC09.NHX7/SOS1* (*BnaC09G0255900ZS*), co-enriched in “ROS metabolic process” (GO:0072593), synergize to mitigate oxidative stress. RCD1 modulates ROS signaling or scavenging, while *NHX7/SOS1*, a Na^+^/H^+^ antiporter, maintains ion homeostasis by extruding cytotoxic Na^+^, indirectly regulating ionic homeostasis [30]. These interconnected pathways—JA signaling amplification, developmental arrest under stress, trehalose-driven osmotic protection, and ROS-ion homeostasis co-regulation—collectively underpin hierarchical salt tolerance strategy in S268, indicating their roles in salt tolerance.

### 2.4. Identification of Differentially Expressed miRNAs (DEmiRNAs) and Their Target Genes Under Salt Stress

**Figure 6:**
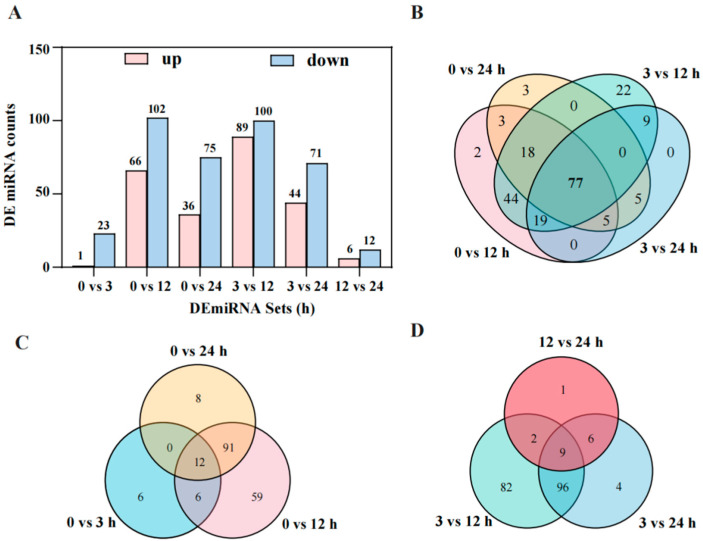
Differential expression analysis of salt-responsive miRNAs in S268. (**A**) Dynamic changes in the number of upregulated (red) and downregulated (blue) miRNAs during salt stress progression (0, 3, 12, 24 h). (**B**) Venn diagram of core salt-responsive miRNAs shared among four critical comparison groups (0 vs. 12, 0 vs. 24, 3 vs. 12, 3 vs. 24 h). (**C**) Overlapping miRNAs associated with sustained stress response in longitudinal comparisons across time points (0 vs. 3, 0 h vs. 12, 0 vs. 24 h). (**D**) Stage-common miRNAs identified through phase-contrast comparisons (3 vs. 12, 3 vs. 24, 12 vs. 24 h).

This study performed miRNA sequencing (miRNA-seq) analysis of S268 under salt stress, and all data passed stringent quality control. Each sample yielded over 9.90 M clean reads (Table S6). Distinct global miRNA expression patterns were observed across time points (0, 3, 12, and 24 h) (Figure S2). Cluster analysis of differentially expressed miRNAs (DEmiRNAs) revealed that miRNAs at 0 h and 3 h shared similar expression profiles, whereas those at 12 h and 24 h exhibited distinct characteristic patterns (Figure S3). Under salt stress treatment (0–24 h), a total of 625 DEmiRNAs were identified in the *Brassica napus* S268. During the early phase (3–12 h) of salt stress, the number of differentially expressed miRNAs peaked at 189 (89 upregulated and 100 downregulated), representing 31.2% of all DEmiRNAs. Time-series comparative analysis demonstrated significant enrichment of DEmiRNAs in four comparison groups: 0 vs. 12, 0 vs. 24, 3 vs. 12, and 3 vs. 24 (Figure 6A), suggesting these miRNAs may serve as key regulatory nodes in salt stress response (Table S7). A total of 300 miRNAs were identified, including 229 novel miRNAs and 71 known miRNAs. Length distribution characteristics of known and novel miRNAs were visualized (Figure S4 and Table S8). For miRNA target prediction, 268 out of 300 miRNAs were successfully associated with target genes. Notably, all known miRNAs (100%) showed complete target prediction coverage, while 197 novel miRNAs (85.98%) were linked to targets (Table S9). Expression pattern analysis of these miRNAs revealed that novel miRNAs exhibited lower expression thresholds than known miRNAs (Figure S5 and Table S10).

Comparative analysis of time-series DEmiRNAs under salt stress identified 77 common miRNAs shared among the four comparison groups (0 vs. 12, 0 vs. 24, 3 vs. 12, and 3 vs. 24 h) (Figure 6B). Among them, bna-miR172a, bna-miR166f, bna-miR167c, bna-miR167b, and bna-miR403 showed significant changes under different salt stress treatments (Figure S6A). Further analysis showed 12 shared miRNAs across three comparison groups (0 vs. 3, 0 vs. 12, and 0 vs. 24 h) in sustained stress response (Figure 6C), indicating their potential involvement in continuous salt stress adaptation. Among them, bna-miR1140 and bna-miR6034 showed a gradual decrease in expression levels as the duration of salt stress treatment (Figure S6B). Notably, stage-specific analysis revealed nine miRNAs shared among three comparison groups (3 vs. 12, 3 vs. 24, and 12 vs. 24 h) (Figure 6D), suggesting their important roles in mediating posttranscriptional regulation of salt tolerance. In particular, the bna-miR172a showed significant changes in expression levels as the duration of salt stress treatment increased (Figure S6C). The study revealed that the known miRNAs, namely bna-miR172a, bna-miR1140, and bna-miR6034, appeared in at least two comparison groups, indicating their important roles in regulating the salt stress response in *B. napus* S268 (Table S10). Based on these findings, this study integrated 77 core response miRNAs, 12 sustained-response miRNAs, and nine stage-specific miRNAs (a total of 93 miRNAs) as the foundation for subsequent DEmiRNA analysis, facilitating the elucidation of dynamic regulatory mechanisms of miRNAs during salt stress responses.

### 2.5. Functional Characterization of Salt-Responsive miRNA-mRNA Interactions Under Salt Stress

**Figure 7:**
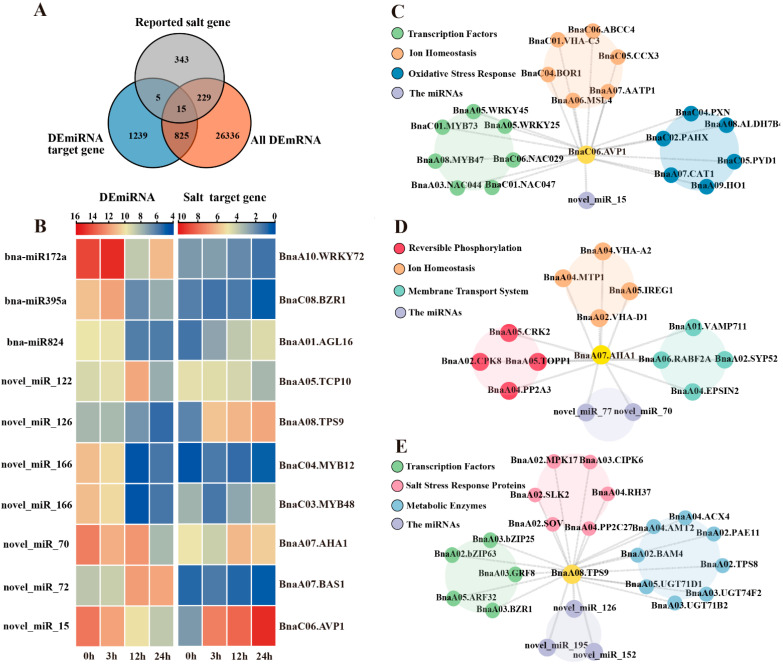
Analysis of the miRNA-mRNA regulatory network in salt stress response of *Brassica napus* S268. (**A**) Screening of salt stress-responsive target genes of differentially expressed miRNAs (DEmiRNAs). (**B**) Analysis of time-series co-expression patterns between DE miRNAs and their target genes. (**C**) Novel_miR15-*BnaC06.AVP1*-mRNA regulatory network. (**D**) Novel_miR70-*BnaA07.AHA1*-mRNA regulatory network. (**E**) Novel_miR126-*BnaA08.TPS9*-mRNA regulatory network.

Following the intersection of target genes from the 93 DEmiRNAs identified by miRNA-seq and all DEGs from transcriptome data, 15 common candidate miRNAs/genes were obtained (Figure 7A). Further analysis revealed 10 regulatory relationships between DEmiRNAs and salt-responsive target genes, including the three known miRNAs (bna-miR172, bna-miR395, and bna-miR824) (Figure 6B). Gene expression pattern analysis demonstrated that five miRNA-mRNA pairs, bna-miR172/*BnaA07.CLV1*, bna-miR395/*BnaC08.BZR1*, novel-miR-126/*BnaA08.TPS9*, novel-miR-15/*BnaC06.AVP1*, and novel-miR-70/*BnaA07.AHA1* exhibited a clear negative trend: the expression levels of miRNAs progressively decreased with prolonged salt stress exposure, while their corresponding target mRNAs showed significant upregulation under the same conditions (Figure 6B). This negative correlation strongly suggests that these miRNAs may directly regulate their target mRNAs or play a dominant regulatory role in salt stress adaptation.

Using WGCNA-based gene co-expression analysis and miRNA–target regulatory relationships, a predictive DEmiRNA-mRNA regulatory network was constructed for three core salt-responsive genes: *BnaA08.TPS9*, *BnaC06.AVP1*, and *BnaA07.AHA1* (Figure 7C–E). The mRNAs interacting with BnaC06.AVP1 were functionally clustered into three categories (Figure 7C): transcription factors, ion homeostasis, and oxidative stress response. The first category comprised genes associated with ion transport and vacuolar acidification, including vacuolar ATPases *(BnaC06.AATP1*, *BnaA07.AATP1*, *BnaC01.VHA-C3*), cation transporters (*BnaC05.CCX3*, *BnaC04.BOR1*), mechanosensitive ion channels (*BnaA06.MSL4*), and ABC transporters (BnaC06.ABCC4). These genes are directly involved in transmembrane ion transport across the vacuolar membrane or proton pump activity. The second category consisted of oxidative stress-responsive genes, such as catalase (*BnaA07.CAT1*), peroxidase (*BnaC04.PXN*), heme oxygenase (*BnaA09.HO1*), and fatty acid α-oxidase (*BnaC02.PAHX*), all functionally linked to reactive oxygen species (ROS) scavenging and detoxification pathways [14,27]. The third category encompassed signaling-related transcription factors, including *NAC* (*BnaC06.NAC029*, *BnaC01.NAC047*, *BnaA03.NAC044*), *WRKY* (*BnaA05.WRKY45*, *BnaA05.WRKY25*), and *MYB* (*BnaA08.MYB47*, *BnaC01.MYB73*), all annotated as stress response regulators.

Phosphorylation-dependent regulation of ion transport and membrane trafficking emerged as core mechanisms underlying the activity of *BnaA07.AHA1* and its salt stress response (12 h MEskyblue3 module). Therefore, mRNAs interacting with *BnaA07.AHA1* were functionally categorized into three groups: reversible phosphorylation, ion homeostasis, and membrane/vesicular transport (Figure 7D). The activity of AHA1 was highly dependent on phosphorylation modifications, particularly at its C-terminal autoinhibitory domain. Protein phosphatases (*BnaA05.TOPP11*, *BnaA04.PP2A3*) and kinases *(BnaA02.CPK8*, *BnaA05.CRK2*) directly or indirectly modulated its proton pump activity, enabling rapid responses to ionic imbalance under salt stress. A central challenge during salt stress involves Na^+^ toxicity and disrupted ion homeostasis. AHA1 coordinated with vacuolar H^+^-ATPases (*BnaA04.VHA-A2* and *BnaA02.VHA-D1*) to regulate transmembrane proton gradients, thereby energizing Na^+^/H^+^ antiporters for Na^+^ efflux. The functionality of AHA1 depended on its dynamic plasma membrane localization. Salt stress potentially regulated AHA1 membrane abundance or activity through Rab GTPases *BnaA06.RABF2A*, SNARE proteins BnaA02.SYP52, and vesicular trafficking components *BnaA01.VAMP711* [16,17,18]. Additionally, clathrin-mediated endocytosis via *BnaA04.EPSIN2* might provide negative feedback to fine-tune AHA1 activity.

Interactome-based predictive analysis revealed that mRNAs (novel_miR_126 and novel_miR152) interacting with *BnaA08.TPS9* exhibited significant enrichment in three functional modules (Figure 7E). The first module comprises transcription factors, including growth regulators such as *BnaA03.GRF8*, BR signaling core factors *BnaA03.BZR1*, auxin response factor *BnaA05.ARF32*, and bZIP family members (*BnaA02.bZIP63* and *BnaA03.bZIP25*), all annotated as direct regulators of stress-responsive or metabolism-associated genes [25]. The second module encompasses salt stress response proteins, notably calcium signaling kinases *BnaA03.CIPK6*, MAP kinase *BnaA02.MPK17*, serine/threonine kinase *BnaA02.SLK2*, protein phosphatase *BnaA04.PP2C27*, and salt overly sensitive protein *BnaA02.SOV*, which collectively mediate salt-responsive signal transduction, ion homeostasis regulation, and RNA metabolism [28]. The third module consists of metabolic enzymes, specifically glycosyltransferases (*BnaA05.UGT71D1*, *BnaA03.UGT71B2*, and *BnaA03.UGT74F2*), the trehalose-6-phosphate synthase *BnaA02.TPS8*, and the ammonium transporter *BnaA04.AMT2*, functioning synergistically in osmoprotectant biosynthesis, membrane lipid repair, and cell wall remodeling.

### 2.6. Validation of RNA-Seq and miRNA-Seq by RT-qPCR

**Figure 8:**
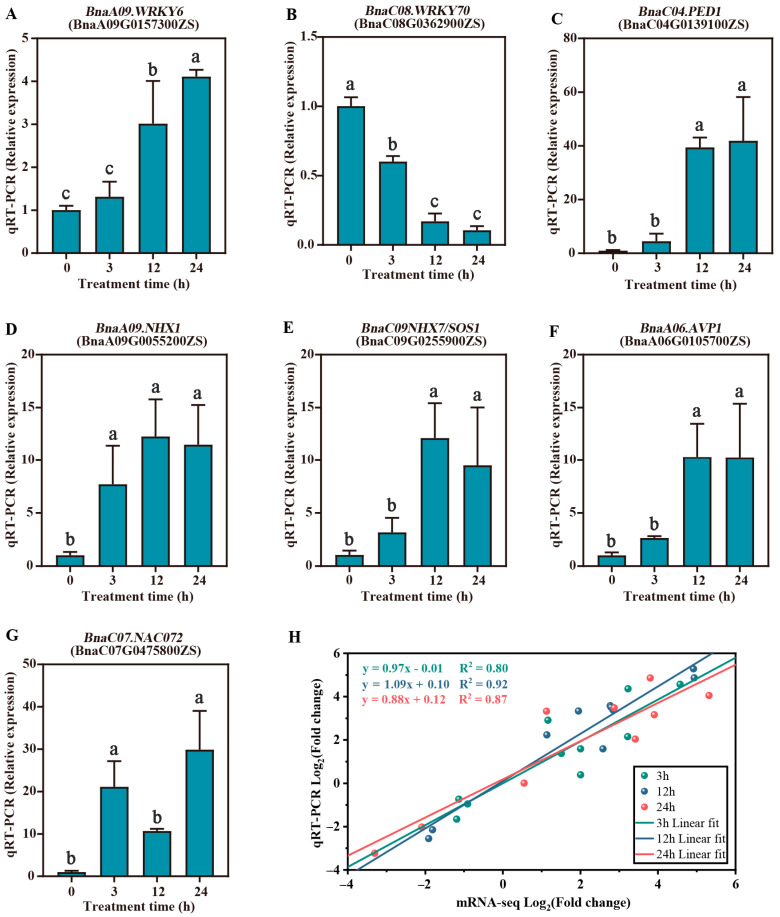
RT-qPCR validation analysis of seven randomly selected differentially expressed genes (DEGs) in response to salt stress. (**A**) *BnaA09WRKY6* (*BnaA09G0157300ZS*); (**B**) *BnaC08. WRKY70* (*BnaC08G0362900ZS*); (**C**) *BnaC04.PED1* (*BnaC04G0139100ZS*); (**D**) *BnaA09. NHX1* (*BnaC09G0055200ZS*); (**E**) *BnaC09. NHX7*/*SOS1* (*BnaC09G0255900ZS*); (**F**) *BnaA06.AVP1* (*BnaA06G0105700ZS*); (**G**) *BnaC07.NAC072* (*BnaC07G0475800ZS*). (**H**) Scatter plot showing Pearson’s correlation of expression change by RT-qPCR (X-axis) and mRNA-seq (Y-axis). Error bars in RT-qPCR detection results represent the standard error (SD) of the mean (*n* = 3). Lowercase letters indicate significant differences at *p* < 0.05 by Student’s *t*-test.

To verify the RNA-seq expression data, we randomly selected seven genes in response to the salt stress for RT-qPCR (Figure 8). Most of these DEGs were induced to up-regulation of the expression level, including the *SOS1* (*BnaC09G0255900ZS*), *WRKY6* (*BnaA09G0157300ZS*), and *NAC072* (*BnaC07G0475800ZS*) (Figure 8A,E,G). Another transcription factor, *WRKY70* (*BnaC08G0362900ZS*), was downregulated in response to the salt stress (Figure 8B). Linear regression analysis demonstrated strong consistency between RT-qPCR validation data and RNA-seq results across all salt stress time points (3, 12, and 24 h). The fitted regression lines for each time point exhibited high linear correlation coefficients (R^2^ > 0.8) (Figure 8H), indicating a robust agreement between the two experimental methodologies.

**Figure 9:**
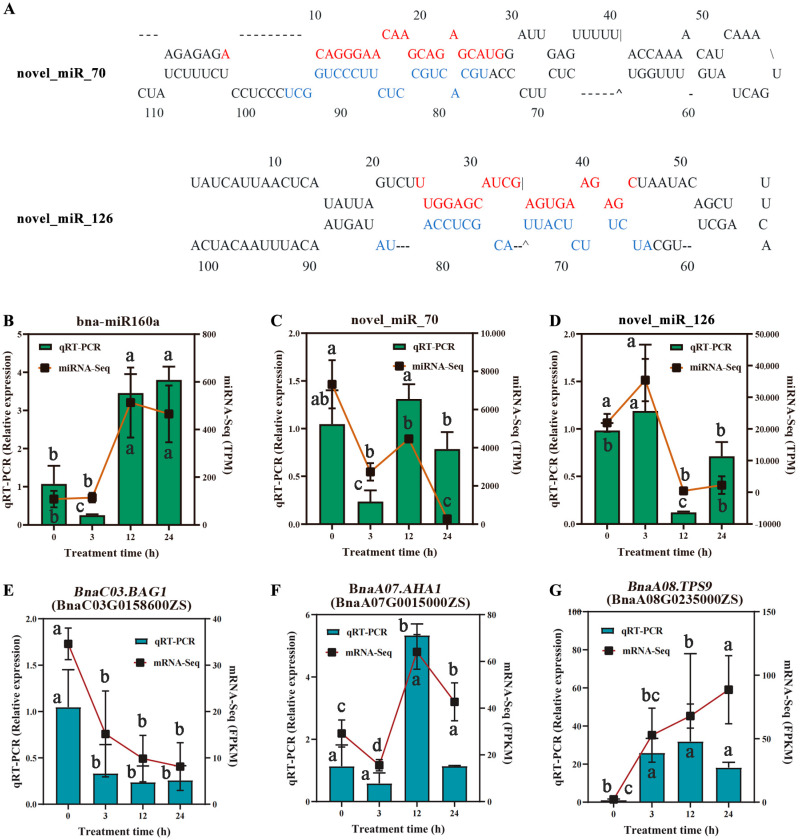
Validation analysis of miRNA-seq and mRNA-seq data using RT-qPCR. (**A**) Predicted stem-loop structures of novel miRNA precursors (novel_miR_70 and novel_miR_126). Red bases represent the mature miRNA sequences, while blue bases indicate the corresponding star (miRNA*) sequences. (**B**,**C**) Correlation analysis between miRNA-seq (TPM-normalized) and RT-qPCR for the relative expression levels of three randomly selected miRNAs. (**D**–**G**) Correlation analysis between mRNA-Seq (FPKM-normalized) and RT-qPCR for the expression levels of the target genes (DEGs) for the three randomly selected mRNAs. Error bars in RT-qPCR detection results represent the standard error (SD) of the mean (*n* = 3). Lowercase letters indicate significant differences at *p* < 0.05 by Student’s *t*-test.

To verify the differential expression patterns of the DEmiRNAs with their target genes, the RT-qPCR was also performed for three random miRNAs (bna-miR160a, novel_miR_70, and novel_miR_126). Similar expression trends were shown for the selected miRNA, suggesting that the miRNA-seq data were reliable (Figure 9). Meanwhile, the two miRNAs (bna-miR160a and novel_miR_126) confirmed the negative association of expression levels of their target genes (*BnaC03.BAG1* and *BnaA08.TPS9*).

## 3. Discussion

Elucidating salt tolerance mechanisms and breeding high-salt-tolerant rapeseed cultivars are vital for expanding its cultivation in saline areas and sustainable agricultural development [31]. However, fewer studies have been reported on the involvement of miRNAs in salt tolerance in rapeseed. Integrative mRNA-miRNA analyses can identify the key genes in regulatory networks in plant development and response to abiotic stresses [32]. In this study, based on the evaluation of 88 rapeseed accessions and physiological results, S268 was identified as the cultivar with a relatively high salt tolerance. The dynamic transcriptomic profiling of S268 revealed dynamic expression patterns of DEGs, suggesting cumulative transcriptional reprogramming under salt stress. Notably, the 212 core DEGs persistently expressed across all time points were enriched in organic acid catabolism and glyoxylate/dicarboxylate metabolism pathways critical for carbon flux rerouting under ionic imbalance [33]. For instance, the suppression of glyoxylate cycle genes like *BnaA08.SHM4* (33.3-fold downregulated) likely redirects carbon toward gluconeogenesis, a strategy observed in salt-tolerant *Arabidopsis* mutants [34]. Conversely, the sustained induction of branched-chain amino acid degradation genes (*BnaC04.PED1*, 72.4-fold upregulated) may fuel the TCA cycle to sustain ATP-dependent ion transport, mirroring metabolic adaptations in halophytic species [33].

Based on the aforementioned DEG analysis results, we further employed WGCNA to explore the co-expression patterns among genes and their potential functional modules for salt tolerance in S268. The WGCNA analysis delineated three temporal regulatory modules (MEdarkorange2, MEskyblue3, and MEivory), each governing distinct phases of salt adaptation (Figure 3A). The early 3 h module (MEdarkorange2) activates ABA biosynthesis and carotenoid metabolism (Figure 4A,B), which is consistent with previous studies that ABA initiates osmotic regulation through stomatal closure and compatible solute synthesis [35,36]. These results suggest that ABA signaling may also play an important role in the salt tolerance of rapeseed S268. The 12 h module (MEskyblue3) emphasized SNARE-mediated vesicle fusion (Figure 4C,D), a mechanism critical for vacuolar Na^+^ sequestration and peroxisomal ROS detoxification, as demonstrated in salt-stressed tomato (*Solanum lycopersicum* L.) [37]. The late 24 h module (MEivory) highlighted SA-JA crosstalk (Figure 4E,F), consistent with reports that SA-JA synergy enhances antioxidant capacity in prolonged salt stress [38]. The persistent upregulation of *BnaC05.TIFY6B* and *BnaC09.NHX7/SOS1* underscores the integration of hormonal signaling and ion homeostasis, a hallmark of elite stress-adapted cultivars (Figure 4 and Figure 8).

Integrative miRNA-mRNA analysis revealed stage-specific regulatory nodes, particularly the bna-miR395/*BnaC08.BZR1*, novel-miR-126/*BnaA08.TPS9*, and novel-miR-15/*BnaC06.AVP1* pairs (Figure 7C,E). The inverse correlation between miRNA downregulation and target mRNA induction aligns with established post-transcriptional silencing mechanisms in plant stress responses [39]. Brassinosteroids (BRs) have been reported to play an important role in abiotic stresses and can alleviate the adverse effects of salinity. In wheat, the BR signal core transcription factor TaBZR1 was demonstrated to activate the ABA biosynthesis gene *TaNCED3* and ROS-scavenging genes *TaGPX2* and *TaGPX3,* improving salt tolerance [21]. In this study, the negative expression patterns of bna-miR395 and its target gene *BnaC08.BZR1* suggested that bna-miR395 might be involved in salt tolerance by rapeseed (Figure 6B).

In soybean, miR172a-AP2/*ERF* has been reported to regulate *TH1* or *RD22*, while *NCED3* has been reported to promote salt tolerance [40]. gma-miR172c-NNC1 could be involved in root tolerance to salt stress, suggesting that miR172 might contribute to salt tolerance via different target genes through different pathways [26]. In our study, miR172a targeted many genes, including *AP2/ERF* (*BnaA07g12050D*), *NAC44* (*BnaC03g32420D*), and *WRKY72* (*BnaA10G0211300ZS*). These miR172a-mRNA pairs might be involved in salt tolerance (Figure 7B and Table S9).

In *Arabidopsis*, *AtTPS9* enhances salt tolerance by promoting suberin lamellae deposition in the root endodermis, thereby reducing Na^+^ translocation to the leaves. This function is mediated through the regulation of the ABA signaling pathway and *SnRK2s* genes. Interestingly, a similar regulatory mechanism may exist in *Brassica napus* S268, where novel-miR-126 is predicted to target *BnaA08.TPS9*, a putative functional homolog of *AtTPS9*. This suggests a potential role for *TPS9* and its upstream regulatory elements in modulating suberin biosynthesis and maintaining ion homeostasis in rapeseed under salt stress conditions [36]. Meanwhile, *BnaA08.TPS9* is predicted to interact with *BnaA03.BZR1*, indicating that the novel miR-126/*BnaA08.TPS9* might be involved in ROS scavenging [21].

In *Brassica napus*, *BnaC06.AVP1,* a functional ortholog of *Arabidopsis AVP1*, was identified to enhance salt tolerance by mediating vacuolar Na^+^ sequestration, a mechanism dependent on its H^+^ pyrophosphatase activity. The novel miR-15 was predicted to target the 3′ UTR of *BnaC06.AVP1*, suggesting its role in post-transcriptional repression via mRNA degradation or translational inhibition. In poplar, miR319a mediated salt tolerance by increasing the lumen area, thereby promoting ion transport [25]. Thus, salt stress may suppress novel miR-15 expression, thereby relieving its inhibitory effect on *BnaC06.AVP1* and establishing a feedback loop to amplify vacuolar ion compartmentalization under stress conditions [41].

This study showed that the miRNA-mRNA pairs play a critical role in salt tolerance in rapeseed. Further functional validation of the candidate genes and miRNAs using the CRISPR-Cas9 system will provide valuable insights into the elucidation of the molecular mechanisms underlying salt tolerance and offer useful resources for breeding salt-resistant rapeseed.

## 4. Materials and Methods

### 4.1. Plant Materials and Salt Stress Treatments

To identify salt-tolerant rapeseed accessions, this study assessed the salt tolerance phenotypes of 88 rapeseed accessions. The rapeseed variety S268 and 87 other rapeseed accessions were sourced from the College of Agronomy, Northwest A&F University. Both control group (distilled water) and experimental group (215 mM NaCl) were established, with three biological replicates for each treatment. Thirty healthy, uniformly sized seeds from each variety were placed in germination dishes lined with moist double-layered filter paper. The control group was treated with 10 mL of distilled water, while the experimental group was subjected to salt stress using 10 mL of 215 mM NaCl solution. The germination dishes were then placed in a rapeseed growth chamber maintained at 54% relative humidity and a temperature range of 18–24 °C, under a 16 h/8 h light/dark cycle. Subsequently, phenotypic measurements were conducted for the 88 accessions during the germination stage. A total of 100 seeds were used for each sample. On the seventh day, both the number of germinated seeds and the number of normal seedlings were recorded. Germination rate, seedling rate, and the relative salt damage index were then calculated. Descriptive statistical analysis and Kolmogorov–Smirnov normality test were conducted on the phenotypic data using R (v 4.4.1).

Relative salt damage index (%) = (Germination rate of control − Germination rate under treatment)/Germination rate of control × 100

### 4.2. Determination of Physiological and Biochemical Indicators

Peroxidase (POD) activity was measured according to the method described in [42]. Briefly, 0.25 g of fresh leaf tissue was homogenized in 2 mL of phosphate buffer (pH 7.8), and the volume was then adjusted to 5 mL. After centrifugation, the supernatant was collected for enzymatic analysis, and absorbance was measured at 470 nm. Proline content was determined using the ninhydrin colorimetric method described by Moore and Stein (1951) [43]. A 0.3 g leaf sample was ground with 3% sulfosalicylic acid and boiled in a water bath for 10 min. After centrifugation, the absorbance of the supernatant was measured at 520 nm. Soluble protein content was quantified following the method of Kielkopf et al. (2020) [44], with absorbance recorded at 595 nm. Total chlorophyll content was determined spectrophotometrically by extracting pigments from fresh leaves using 80% acetone. Absorbance was measured at 645 nm and 663 nm, and the total chlorophyll concentration was calculated using standard formulas [45].

### 4.3. Preparation of Plant Materials for mRNA-Seq and miRNA-Seq

To minimize the impact of extraneous factors in the plant growth environment on sequencing outcomes, this study employed a hydroponic cultivation approach for rapeseed to prepare NGS samples. Initially, 20 uniform and healthy S268 seeds were sterilized. These seeds were then placed in a Petri dish lined with double-layer moist filter paper and incubated at 4 °C for 24 h, followed by a six-day growth period in a rapeseed growth chamber. Seedlings exhibiting uniform growth were subsequently transferred to a 10 × 15 planting float board equipped with sponges. The float board was immersed in a 10 L hydroponic tank filled with a 1/2 strength Hoagland nutrient solution. Upon reaching the three-leaf stage, the nutrient solution was upgraded to full strength. At the four-leaf stage, the plants were subjected to salt stress by replacing the nutrient solution with a 200 mM NaCl solution. Leaves were sampled at 0, 3, 12, and 24 h post-treatment, specifically the second fully expanded leaf from the base, which was then flash-frozen in liquid nitrogen and dispatched to Biomarker for RNA and sRNA sequencing. Each treatment condition was replicated at least twice to ensure biological reproducibility.

### 4.4. Construction and Sequencing of mRNA-Seq and miRNA-Seq Libraries

Total RNA was isolated from rapeseed leaves with TRIzol reagent (Invitrogen, Carlsbad, CA, USA), followed by quantification and quality assessment using a Nanodrop 2000 (Thermo, Waltham, MA, USA). The integrity of the RNA was examined using an Agilent 2100 LabChip GX (Agilent Technologies, Santa Clara, CA, USA). Quality-approved samples were used to construct cDNA libraries using the VAHTS Universal V6 RNA-seq Library Prep Kit for Illumina, and the resulting mRNA libraries were sequenced on the Illumina NovaSeq 6000 platform (Illumina, San Diego, CA, USA) [46] using paired-end sequencing. To generate clean mRNA reads, the raw sequencing data were processed using fastp (v0.23.4). Adapter sequences were automatically detected and removed. Reads with more than 50% low-quality bases (Phred < 10) or more than 10 ambiguous bases (N) were discarded. Clean paired-end reads were retained for downstream analysis [47]. The resulting high-quality reads were then aligned to the *Brassica napus* ZS11 V0 (https://yanglab.hzau.edu.cn/BnIR/germplasm_info?id=ZS11.v0, accessed on 15 January 2025) reference genome using HISAT2 (v2.0.5) [48]. Gene expression in all twelve libraries was quantified and reported in FPKM (fragments per kilobase of transcript per million mapped reads).

Quality-approved samples were first used to construct cDNA libraries with the VAHTS™ Small RNA Library Prep Kit for Illumina (Vazyme Biotech Co., Ltd., Nanjing, China). The resulting small RNA libraries were sequenced on the Illumina NovaSeq 6000 platform(Illumina, San Diego, CA, USA) [46], generating 50 bp single-end reads. Using Bowtie software (v1.1.1) [49], unannotated sRNA reads were aligned to the *Brassica napus* ZS11 V0 reference genome to obtain their genomic position information. The clean reads were subsequently aligned against the Silva, GtRNAdb, Rfam, and Repbase databases to filter out rRNA, tRNA, snRNA, snoRNA, and other non-coding RNAs, as well as repetitive sequences, resulting in unannotated reads that included miRNAs.

### 4.5. miRNA Identification and Target Gene Prediction

In the identification of known miRNAs, reads aligned to the reference genome were compared with mature miRNA sequences from the miRBase (v22) database, including their flanking regions (2 nt upstream and 5 nt downstream), allowing for a maximum of one mismatch. Reads that matched under these conditions were considered as known miRNAs. miRNA precursors exhibit a characteristic hairpin structure, and the mature miRNA is generated through cleavage by Dicer/DCL enzymes. For sequences that did not match known miRNAs, novel miRNAs were predicted using the miRDeep2 software [50]. miRDeep2 identifies potential precursor sequences based on the genomic locations of the reads, incorporating read distribution features (mature, star, loop) and precursor structural energy information (RNAfold, randfold), and employs a Bayesian model for scoring and prediction [51]. miRNA expression levels were normalized using the TPM (Transcripts Per Million) algorithm [52], calculated as TPM = (Readcount × 1,000,000)/Mapped Reads, where Readcount represents the number of reads aligned to a specific miRNA, and Mapped Reads denotes the total number of reads aligned to all miRNAs. The psRNAtarget tool was utilized to predict mRNAs that serve as target genes for miRNAs [53].

### 4.6. Analysis of the Differential Expression of mRNA and miRNA

For the differential expression analysis of mRNA, we utilized the DESeq2 R package (version 1.10.1) for data processing and statistical analysis [54]. Initially, raw sequencing data were aligned to the reference genome, and a gene expression count matrix was generated using the HTSeq tool. Based on the negative binomial distribution model, DESeq2 normalized the gene expression data and calculated the expression differences of genes between different sample groups. The criteria for screening differentially expressed genes (DEGs) were |log2FoldChange| ≥ 1 and *p*-value < 0.05. To control the false positive rate caused by multiple hypothesis testing, we used the Benjamini–Hochberg method to adjust the *p*-values, resulting in the False Discovery Rate (FDR). Through this analysis, we obtained a list of significantly differentially expressed mRNAs, which were further used for functional annotation and pathway enrichment analysis.

For the differential expression analysis of miRNA, we employed the DESeq R package for statistical analysis. The criteria for screening differentially expressed miRNAs (DEmiRNAs) were |log2FoldChange| ≥ 1 and adjusted *p*-value ≤ 0.05. Through this analysis, we identified significantly differentially expressed miRNAs and further analyzed their potential target genes and biological functions. For the predicted novel miRNAs, secondary structures of potential miRNA precursors were constructed using the MFOLD3.2 web server. After log2 transformation and normalization, the candidate miRNA–mRNA pairs containing DEmiRNAs and DEGs were selected as miRNA–target pairs.

### 4.7. Co-Expression Network Construction

A weighted gene co-expression network of differentially expressed mRNAs was constructed using the WGCNA package in R (v4.4.1) [55]. An unsupervised co-expression relationship was built based on the adjacency matrix, which represents the network connection strength between gene pairs. The one-step network construction option with a soft thresholding power value of 15, min Module Size = 100, and merge Cut Height = 0.2 was used. The other parameters were set to default values, and the q-values (false discovery rate, FDR) were calculated to test the significance (q-value < 0.05). Highly similar modules were subsequently identified by clustering and then merged into new modules based on eigengenes. Correlations between various modules were analyzed and visualized using heatmaps. To identify key genes associated with salt tolerance, an in-depth analysis of mRNA-mRNA interaction relationships was conducted utilizing the STRING protein–protein interaction database. The co-expression network was visualized using Cytoscape software [56].

### 4.8. GO and KEGG Pathway Enrichment Analysis

The specific workflow for GO and KEGG pathway enrichment analysis of differentially expressed genes (DEGs) from mRNA sequencing and target genes of differentially expressed miRNAs is as follows: GO enrichment analysis of the DEGs was performed using the GOseq R package with default parameters, which corrects for gene length bias [57]. GO enrichment analysis was also conducted using the ClusterProfiler R package, which is based on the Wallenius non-central hyper-geometric distribution. For pathway analysis, the KEGG database was utilized to understand high-level functions and utilities of biological systems using KOBAS software (v 2.0) [58].

### 4.9. Haplotype Analysis

Based on mRNA-seq and miRNA-seq analysis, miRNAs and their predicted target genes with significant differential expression under salt stress were screened, and candidate genes were identified. Combined with whole genome resequencing data of 125 rapeseed accessions, the SNP information in the region where they were located was extracted for haplotype construction [7]. All materials were divided into different haplotype types based on the allele combination of the samples at these SNP sites. To ensure the stability of statistical analysis, only haplotype types with a sample number of at least 10 were retained for subsequent analysis.

Phenotypic data were obtained from phenotypic measurements of the same materials under salt stress treatment, including germination rate, seedling rate, and relative salt damage index. One-way ANOVA was used to test the phenotypic differences between different haplotype groups, and Tukey’s HSD test was used for pairwise comparison, with the significance level set at *p* < 0.05.

### 4.10. Validation of mRNA and miRNA Expressions

Total RNA was isolated from each experimental group using TRIzol reagent (Invitrogen, USA) following the previously outlined protocol. Complementary DNA (cDNA) synthesis was carried out with the RevertAid First Strand cDNA Synthesis Kit (ThermoFisher, Shanghai, China) in accordance with the manufacturer’s guidelines. The synthesized cDNA was subsequently diluted tenfold to serve as a template for quantitative PCR (qPCR). Quantitative reverse transcription PCR (RT-qPCR) assays were conducted using SYBR Green Supermix (Takara, Dalian, China) on a QuantStudioTM 7 Flex System. For mRNA quantification, the *BnaActin7* gene was employed as an internal control, while U6 served as the reference gene for stem-loop miRNA analysis [59]. Three independent biological replicates were used for each RT-qPCR experiment. Data are presented as mean ± standard deviation (SD). The relative expression levels of target genes were determined using the 2^−ΔΔCT^ method [60]. The specific primers utilized in the RT-qPCR assays are provided in Table S11.

## 5. Conclusions

Overall, this study elucidates the molecular mechanism underlying the salt tolerance of rapeseed cultivar S268 through integrative mRNA-miRNA transcription profiling. A total of 212 core genes and 93 DEmiRNAs were identified in response to NaCl salt stress in rapeseed S268. In the present study, known miRNAs (bna-miR172, bna-miR395, bna-miR824) and their targets, as well as novel miRNA regulatory pairs (novel-miR-126/*BnaA08.TPS9,* and novel-miR-70/*BnaA07.AHA1*) were uncovered. Most of these miRNAs and targets are involved in abscisic acid (ABA) signaling and SNARE complex-mediated membrane trafficking pathways to regulate salt stress in rapeseed, indicating they might be involved in miRNA-mediated regulatory networks under NaCl salt stress. Future research should use the CRISPR-Cas9 system to validate the identified key candidate genes and miRNAs by generating gene-edited materials and analyzing their salt tolerance phenotypes, thereby providing direct evidence and a better understanding of the molecular mechanisms of salt tolerance in rapeseed. The findings of this study provide insights into miRNA–mRNA regulation in the NaCl stress tolerance of rapeseed, offering a valuable resource for the development of salt-resilient cultivars.

## Figures and Tables

**Table 1:** Functional GO enrichment analysis of salt stress-responsive genes in stage-specific modules of *Brassica napus* S268.

The Module of WGCNA	Gene Name	Gene ID	Category	GO ID	Term	Gene Ratio	*p*-Value
24 h MEivory	TIFY6B	BnaC05G0424600ZS	Biological Process	GO: 2000022	Regulation of the jasmonic acid-mediated signaling pathway	25/1574	3.55 × 10^−9^
24 h MEivory	ABI5	BnaA05G0087100ZS	Biological Process	GO: 0010029	Regulation of seed germination	29/1574	6.58 × 10^−5^
24 h MEivory	ABI5	BnaA05G0087100ZS	Biological Process	GO: 1900140	Regulation of seedling development	29/1574	1.89 × 10^−4^
24 h MEivory	TPS9	BnaA08G0235000ZS	Biological Process	GO: 0070413	Trehalose metabolism in response to stress	10/1574	2.45 × 10^−4^
24 h MEivory	RCD1	BnaC05G0343300ZS	Biological Process	GO: 0072593	Reactive oxygen species metabolic process	28/1574	1.23 × 10^−3^
24 h MEivory	NHX7/ SOS1	BnaC09G0255900ZS	Biological Process	GO: 0072593	Reactive oxygen species metabolic process	28/1574	1.23 × 10^−3^

## Data Availability

The raw sequencing data generated in this study are available in GSA (https://ngdc.cncb.ac.cn/gsa) (accessed on 23 June 2025) of CNCB with the accession number PRJC042087.

## Supplementary Materials

The following supporting information can be downloaded at: https://www.mdpi.com/article/10.3390/plants14152418/s1, Figure S1: Functional enrichment analysis of time-specific WGCNA modules in salt-stressed *B. napus* S268; Figure S2: Dynamic analysis of miRNA expression in rapeseed variety S268; Figure S3: Differentially expressed miRNA clustering heatmap; Figure S4: Identified known miRNAs, novel miRNAs, and overall miRNA length distribution; Figure S5: Expression profiling of 300 salt stress-responsive miRNAs in *B. napus* S268; Figure S6: Analysis of DEmiRNA gene expression patterns in *B. napus* S268; Table S1: Descriptive statistics of salt tolerance phenotypes; Table S2: RNA-Seq information for *B. napus* S268 samples; Table S3: GO enrichment analysis of the 212 core genes common to the six comparison groups in *B. napus* S268; Table S4: KEGG enrichment analysis of the 212 core genes common to the six comparison groups in *B. napus* S268; Table S5: GO and KEGG analysis of the three time-specific modules based on WGCNA; Table S6: miRNA-Seq information for *B. napus* S268 samples; Table S7: The predicted target genes for known miRNAs; Table S8: The predicted target genes for novel miRNAs; Table S9: Target gene prediction of all DEmiRNAs in *B. napus* S268; Table S10: The expression levels of DEmiRNAs in *B. napus* S268; Table S11: Primers used for qRT-PCR in this study.

## Abbreviations

The following abbreviations are used in this manuscript:

ABA	Abscisic acid
BRs	Brassinosteroids
cDNA	Complementary DNA
DEGs	Differentially expressed genes
DEmiRNAs	Differentially expressed miRNAs
FDR	False discovery rate
FPKM	Fragments per kilobase of transcript per million mapped reads
JA	Jasmonic acid
RT-qPCR	Quantitative reverse transcription PCR
ROS	Reactive oxygen species
TPM	Transcripts per million
NGS	Next-generation sequencing
WGCNA	Weighted gene co-expression network analysis
