# Supplementary material for: Integration of mRNA and miRNA Analysis Reveals the Regulation of Salt Stress Response in Rapeseed (Brassica napus L.)
Source: Plants (Basel). 2025 Aug 4;14(15):2418. doi: 10.3390/plants14152418 (PMC12349528; doi:10.3390/plants14152418)
Supplement: Supplementary file 1 [file plants-14-02418-s001.zip › Supplementary Figures.pdf]

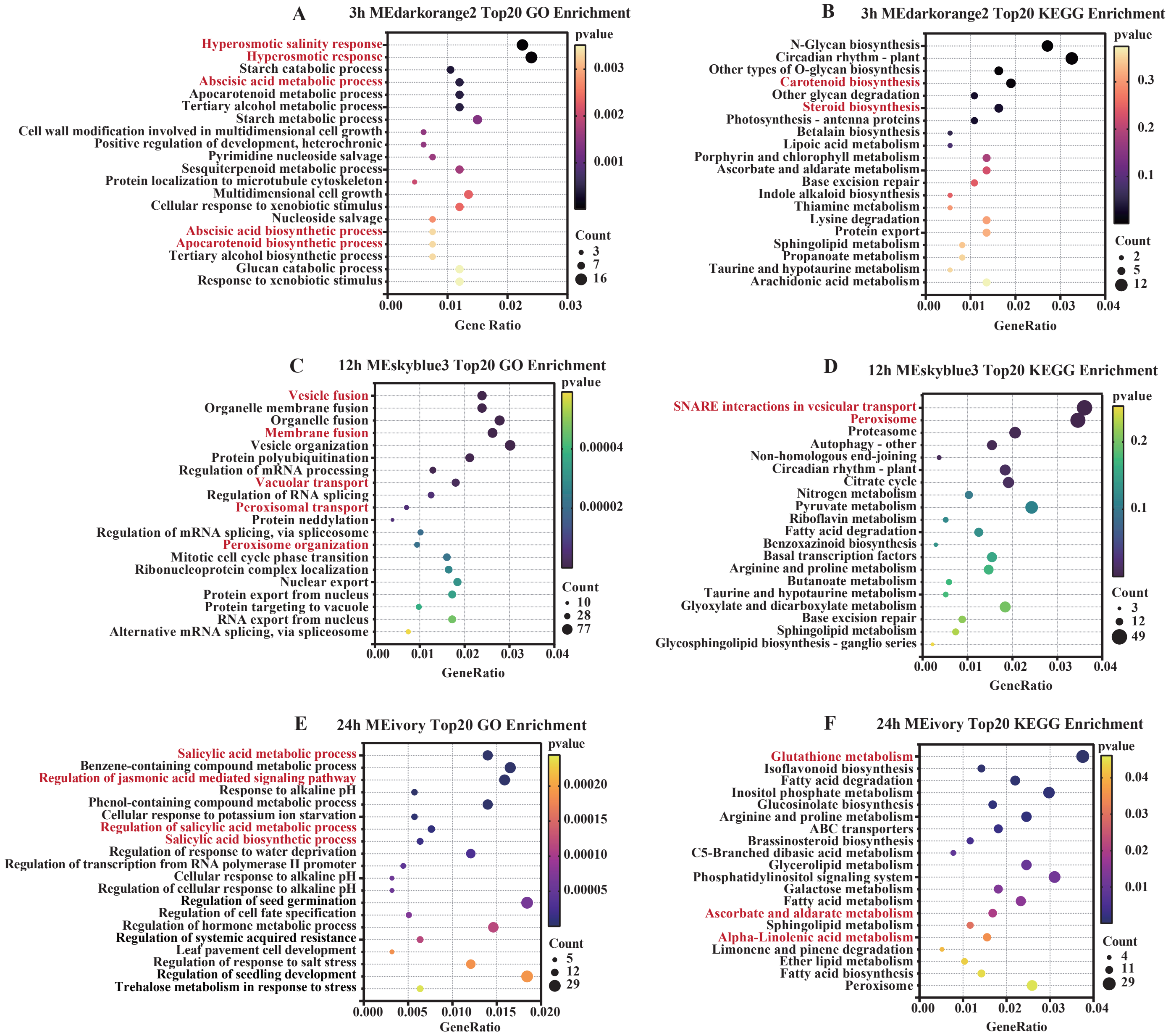

Figure S1 Functional enrichment analysis of time-specific WGCNA modules in salt-stressed *B. napus* S268: (A-B) GO-BP and KEGG pathway enrichment anal-yses of the MEdarkorange2 module associated with 3 h salt stress; (C-D) GO-BP and KEGG enrichment analyses of the MEskyblue3 module linked to 12 h salt stress; (E-F) GO-BP and KEGG enrichment analyses of the MEivory module corresponding to 24 h salt stress.

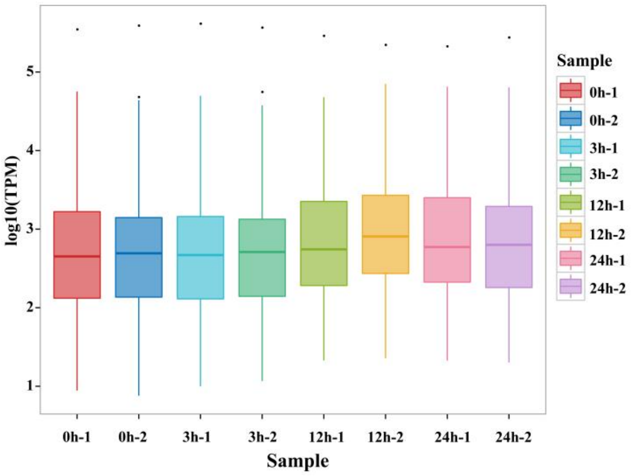

Figure S2 Dynamic Analysis of miRNA Expression in S268 Rapeseed Variety. Each time point includes two biological replicates (labeled as 1 and 2). Data are presented as $\log_{10}{}^{(TPM)}$ values, with distinct colors indicating different samples.

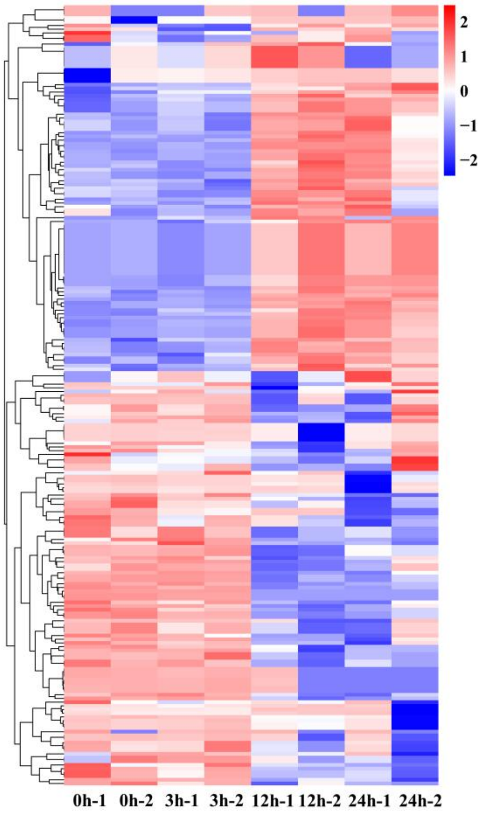

Figure S3 Differentially expressed miRNA clustering heatmap. Columns represent different samples, rows represent different miRNAs, and clustering is performed based on $\log_{10}{}^{(TPM+1E-6)}$ values. Red indicates highly expressed miRNAs, while blue indicates lowly expressed miRNAs.

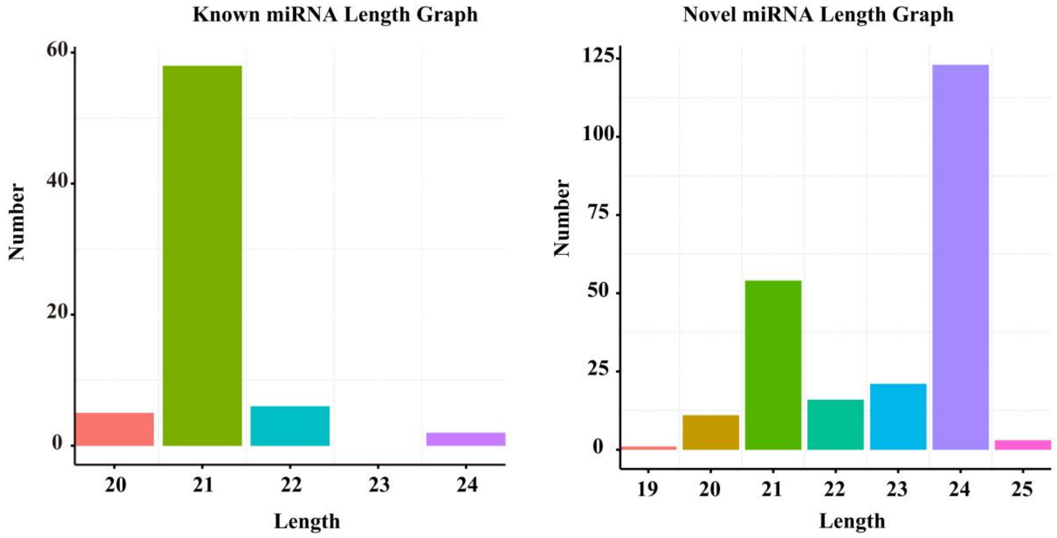

Figure S4 Identified Known miRNAs, Novel miRNAs, and Overall miRNA Length Distribution. The x-axis represents miRNA length. The y-axis represents the number of miRNAs at specific lengths.

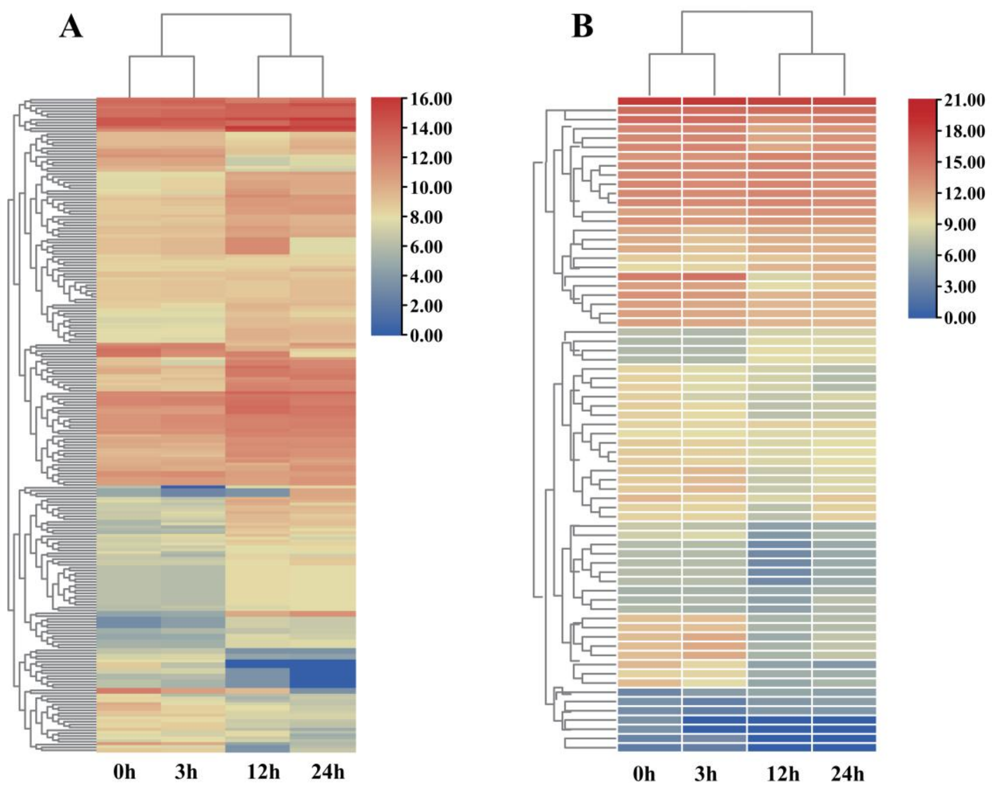

Figure S5 Expression profiling of 300 salt stress-responsive miRNAs in *B. napus* S268: (A) 229 novel miRNAs; (B) 71 known miRNAs.

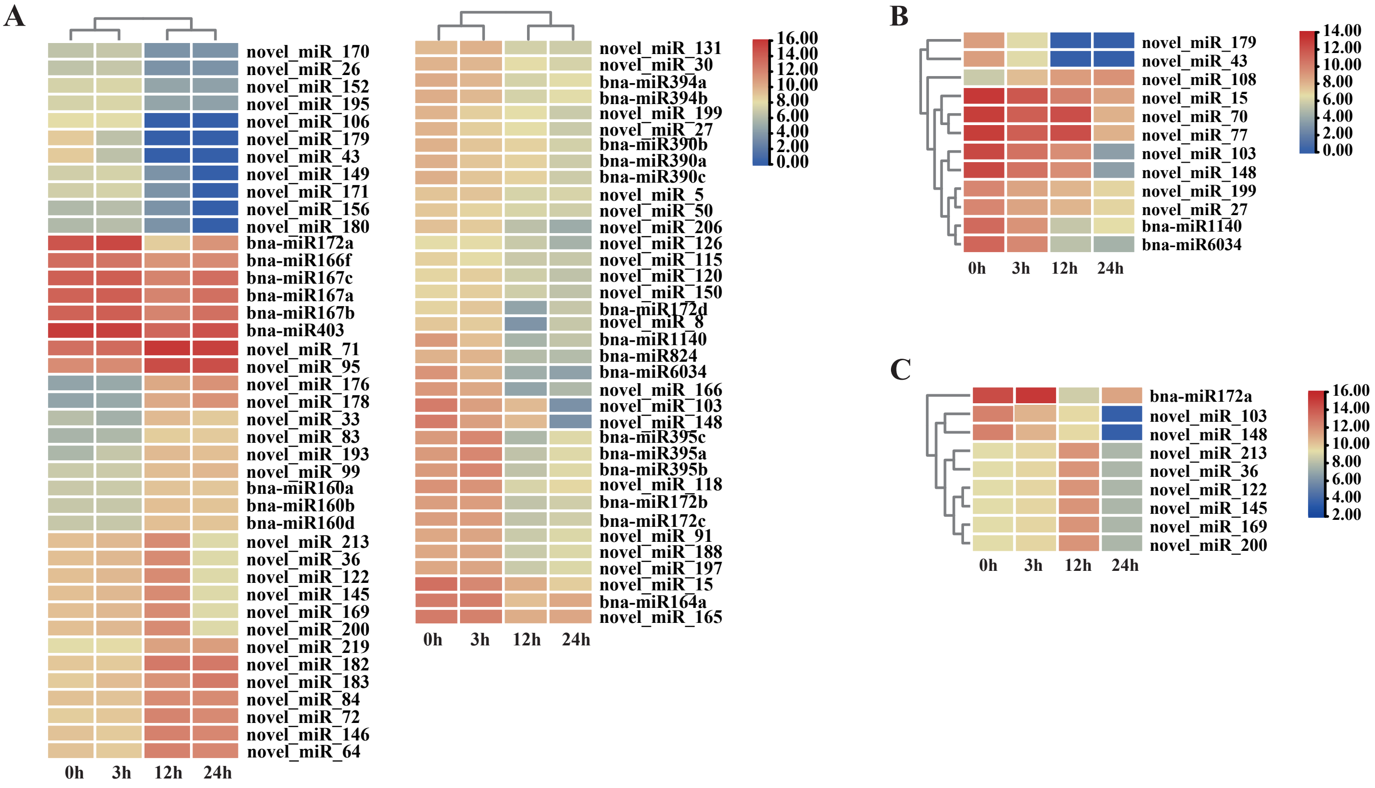

Figure S6 Analysis of DEmiRNA gene expression patterns in *B. napus* S268: (A) 77 common elements in (0 vs 12, 0 vs 24, 3 vs 12, and 3 vs 24 h); (B) 12 common elements in (0 vs. 3, 0 vs 12, and 0 vs 24 h); (C) 9 common elements in (3 vs 12, 3 vs 24, and 12 vs 24 h).
